# Exposure of *Caralluma tuberculata* to biogenic selenium nanoparticles as *in vitro* rooting agent: Stimulates morpho-physiological and antioxidant defense system

**DOI:** 10.1371/journal.pone.0297764

**Published:** 2024-04-10

**Authors:** Amir Ali, Zia-ur-Rehman Mashwani, Naveed Iqbal Raja, Sher Mohammad, M. Sheeraz Ahmad, Juan Pedro Luna-Arias

**Affiliations:** 1 Department of Botany, PMAS Arid Agriculture University Rawalpindi, Rawalpindi, Pakistan; 2 Biotechnology Laboratory, Agricultural Research Institute (ARI) Tarnab, Peshawar, Pakistan; 3 Pakistan Academy of Sciences, Islamabad, Pakistan; 4 University Institute of Biochemistry and Biotechnology (UIBB), PMAS-Arid Agriculture University, Rawalpindi, Pakistan; 5 Department of Cell Biology and Nanoscience and Nanotechnology Ph.D. Program, Center for Research and Advanced Studies of the National Polytechnic Institute, Mexico City, Mexico; ICAR - Central Tobacco Research Institute, INDIA

## Abstract

The commercial-scale production of *Caralluma tuberculata* faces significant challenges due to lower seed viability and sluggish rate of root growth in natural conditions. To overcome these obstacles, using phyto-mediated selenium nanomaterials as an *in vitro* rooting agent in plant *in vitro* cultures is a promising approach to facilitate rapid propagation and enhance the production of valuable therapeutic compounds. This study aimed to investigate the impact of phytosynthesized selenium nanoparticles (SeNPs) on the morphological growth attributes, physiological status, and secondary metabolite fabrication in *in vitro* propagated *Caralluma tuberculata*. The results demonstrated that a lower dose of SeNPs (100 μg/L) along with plant growth regulators (IBA 1 mg/L) had an affirmative effect on growth parameters and promoted earliest root initiation (4.6±0.98 days), highest rooting frequency (68.21±5.12%), number of roots (6.3±1.8), maximum fresh weight (710±6.01 mg) and dry weight (549.89±6.77 mg). However, higher levels of SeNPs (200 and 400 μg/L) in the growth media proved detrimental to growth and development. Further, stress caused by SeNPs at 100 μg/L along with PGRs (IBA 1 mg/L) produced a higher level of total chlorophyll contents (32.66± 4.36 μg/ml), while cultures exposed to 200 μg/L SeNPs alone exhibited the maximum amount of proline contents (10.5± 1.32 μg/ml). Interestingly, exposure to 400 μg/L SeNPs induced a stress response in the cultures, leading to increased levels of total phenolic content (3.4 ± 0.052), total flavonoid content (1.8 ± 0.034), and antioxidant activity 82 ± 4.8%). Furthermore, the combination of 100 μg/L SeNPs and plant growth regulators (1 mg/L IBA) led to accelerated enzymatic antioxidant activities, including superoxide dismutase (SOD = 4.4 ± 0.067 U/mg), peroxidase dismutase (POD = 3.3 ± 0.043 U/mg), catalase (CAT = 2.8 ± 0.048 U/mg), and ascorbate peroxidase (APx = 1.6 ± 0.082 U/mg). This is the first report that highlights the efficacy of SeNPs in culture media and presents a promising approach for the commercial propagation of *C*. *tuberculata* with a strong antioxidant defense system *in vitro*.

## 1. Introduction

*Caralluma tuberculata*, a member of the Apocynaceae family, is a leafless and fleshy plant with medicinal and edible properties. It has angular stems that can grow up near 15 cm, and its branches terminate in dark purple flowers [1]. In different regions of the world, it is known by various local names such as chung, pamanky aputag, and marmootk [2]. *C*. *tuberculata* is rich in bioactive metabolites and exhibits high antioxidant potential. It is commonly used in the treatment of diabetes, inflammation, asthma, paralysis, obesity, joint pains, and fever [3,4].

Regrettably, the *C*. *tuberculata* species has experienced a significant decline in its natural habitat worldwide due to excessive exploitation for pharmaceutical purposes. In Pakistan, the hilly areas of the Hindu Kush and Suleiman regions in the Baluchistan and Khyber Pakhtunkhwa provinces are home to two *Caralluma* species: *C*. *tuberculata* and *C*. *edulis* [5]. However, the viability of *C*. *tuberculata* seeds is low, resulting in a poor germination rate. Consequently, the plant is primarily propagated through the laborious process of stem cuttings. Therefore, there is a pressing need for alternative methods to propagate, produce biomass, and conserve this plant species. Plant *in vitro* cultures present a propitious technology for achieving dramatic enhancement in propagation and the production of phytochemicals within a shorter time frame.

Several precautions can be implemented to enhance plantlets’ quality and resilience during the acclimatization process. One effective measure involves optimizing the culture conditions and incorporating rooting agents to enhance the efficiency of *in vitro* rooting [6]. Additionally, stimulating the plant’s defense machinery through useful biological sources has proven advantageous. Usually, auxins have been employed to endorse *in vitro* rooting by supplementing the growth medium. However, using auxins has certain limitations, including diminished effectiveness after post-autoclaving stages and inhibition of root elongation at high concentrations [7,8]. Likewise, auxins have been found to hinder stomatal growth, negatively impacting tissue-cultured plants [9].

In recent years, the application of nanomaterials has garnered attention as a potential solution to various challenges across different fields. The incorporation of nutritional minerals in the form of nanoparticles (NPs) into plant tissue culture has yielded positive outcomes. For example, copper NPs have been successfully utilized as a replacement for copper sulfate in basil somatic embryogenesis, while silver NPs have been incorporated as a substitute for cobalt chloride in rose culture [10]. Despite the promising advantages of selenium nanoparticles (SeNPs), there is a dearth of extensive research on their effects on plant micropropagation. Selenium contributes to numerous physio-biochemical progressions facilitating growth and enhancing stress tolerance of plants [11–14].

Recognizing the potential benefits of selenium for plants, we conducted a study on the micropropagation of *Caralluma tuberculata* by incorporating Biogenic SeNPs into the culture media as an elicitor. Our objective was to assess the influence of Biogenic SeNPs on the *in vitro* rooting development of *C*. *tuberculata*. Furthermore, we aimed to determine the toxicity threshold of selenium for *Caralluma tuberculata in vitro* cultures. Specifically, we assessed the consequences of SeNPs on the growth development and antioxidant status of *in vitro* plantlets of *C*. *tuberculata*.

## 2. Material and methods

### 2.1. Green synthesis of selenium nanoparticles

The green synthesis of SeNPs was performed in the Department of Botany, PMAS Arid Agriculture University Rawalpindi. For the green synthesis of SeNPs, five grams of garlic cloves were collected and washed with tap water, followed by distilled water. The washed cloves were chopped, forming a fine paste using a porcelain mortar and pestle. Then the garlic clove paste was supplemented with distilled water (400 mL) with continuous stirring and boiled covered with aluminum foil on a hot plate for 20 min. The extract was filtered through a Whatman No. 1 filter paper and kept at 4°C until its use. For the formulation of SeNPs, 20 mL of a 10 mM sodium selenite (Sigma Aldrich) solution was prepared, and the garlic extract (10 mL) was added dropwise under constant magnetic stirring. Then, the solution was retained at 120 rpm under 36°C on a shaker for 4 to 6 days under dark conditions. The formation of SeNPs was demonstrated by observing a color change from uncolored to brick red. The mixture was centrifuged for 15 minutes at 10,000 rpm, room temperature, and the supernatant was discarded; the pellet was resuspended in 2 mL methanol, and centrifuged as mentioned and the pellet containing the purified SeNPs was dried and stored for experimentation [15].

### 2.2. Physicochemical characterization of selenium nanoparticles

After visual observation, the initial formation of plant-based SeNPs has been confirmed through UV visible spectrometry. The sample was prepared by immersing the SeNPs in distilled water and sonicating it for 15 minutes. The absorbance spectrum was noted from 200 to 700 nm through a spectrophotometer. FTIR spectrometry was used to determine the functional groups in the SeNPs. Characterization was performed in the 400–4000 cm^-1^ wave number range with an FTIR spectrometer (NICOLET 6700, Thermo, Waltham, MA, USA).

Structure characterization of SeNPs was done through Scanning Electron Microscopy (SEM, JSM5910 JEOL, Tokyo, Japan). The SEM’s magnification was adjusted to 10 k, and the scanning electron was set at 5kV. The sample was prepared through the drop procedure, which makes use of a copper grid that has been carbon-coated. Samples were dropped onto a copper-coated grid to create a film of SeNPs. The excess solution was blotted out with blotting paper, and the film was dried for ten minutes under a mercury lamp. At different magnifications, the surface topography of SeNPs was examined.

EDX detector (SIGMA model) was used to analyze the elemental composition of photosynthesized SeNPs by using the previous protocol [16].

### 2.3. Establishment of culture conditions for in vitro plantlet induction

To optimize the conditions for *in vitro* plantlet induction, three various doses of Indole-3-butyric acid (IBA) 0, 0.5, 1, 1.5, 2, 2.5, and 3mg/L were tested along with three different types of explants: small shoot (1.5 cm), large shoot (3 cm), and cut shoot (1 cm). The growth medium consisted of MS [17] medium supplemented with 3% sucrose and IBA. The growth medium pH was attuned to 5.8, and agar was supplemented at a concentration of 7 g/L preceding to autoclave (121°C) for 20 minutes. The explants were collected from potted plants, subjected to surface sterilization, and inoculated on MS media under a sterile environment and kept in a growth room at 25 ± 1°C, relative humidity of 70%, light intensity (40–50 μmol/m2/s) and photoperiod i.e 16 hours of light and 8 hours of darkness.

#### 2.3.1. Effect of Phyto-mediated SeNPs on morphological growth features of *C*. *tuberculate*

To scrutinize the effect of selenium nanoparticles (SeNPs) on the growth parameters of *C*. *tuberculata*, a phytosynthesized SeNPs nanoparticle solution was prepared in distilled water [18]. To check the influence of SeNPs, plants established on growth medium contained IBA (1 mg/L) and small shoots (1.5 cm) as the explant source were chosen as the control group. The growth media consisted of MS medium with sucrose (30 g/L), IBA (1 mg/L), and agar (7 g/L). After pH adjustment (5.8), and sterilization of the media, all the tubes were shifted to a laminar airflow hood. Different concentrations of SeNPs (50, 100, 200, and 400 μg/L) were added to the media, either alone or in combination with IBA. The SeNPs were properly dispersed to prevent agglomeration, and the media (7 ± 1 mL) were distributed in sterilized test tubes. The explants (small shoots) were cultured under a controlled environment. Various growth parameters such as root initiation, percent response of roots, root quantity, and fresh and dry weight were recorded after four weeks [19].

### 2.4. Physiological attributes

The physiological characteristics of *in vitro Caralluma tuberculata* plantlets were assessed by determining the total chlorophyll content and total proline content. For the determination of total chlorophyll content, 0.2 g of plant tissue was ground in a cold 80% acetone solution, and the absorbance was measured at 652 nm using the method proposed by [20].

The method described by [21] was followed for proline content determination. 0.2 g of leaf tissue was crushed in 10 mL of sulfosalicylic acid to determine the proline content. The mixture was filtered through Whatman filter paper no.1 and then centrifuged at 10,000 rpm for 10 minutes. In a test tube, 2 mL of the filtrate was combined with 2 mL of glacial acetic acid and 2 mL of ninhydrin. The mixture was incubated at 100°C in a water bath for 1 hour. Afterward, the test tubes were placed in a desiccator, and 4 mL of toluene was added to each tube. The solution was thoroughly mixed on a vortex for 5 minutes. The upper translucent layer was separated from the two layers of solution, and the absorbance at 520 nm was measured.

### 2.5. Preparation of in vitro grown cultures of C. tuberculata plantlet extraction for phytochemical screening

To assess the elicitors’ effect on bioactive secondary metabolite accumulation, *in vitro* plantlets of *Caralluma tuberculata* were employed for antioxidant examination. All the developed cultures, either treated or untreated with SeNPs, were used. [22] proposed methodology was followed with minor amendments for extracting phytochemicals from samples. In the experiment, from each sample, about 300 mg powder was weighed and dissolved in 10 mL methanol (50%), shaken (24 rpm; 25± 1°C) for 24 hours, sonicated for 30 minutes, followed by vortexing for 5 minutes, and vigorously stirred for 15 minutes. Afterward, the resulting samples were centrifuged at 6,500 rpm for 10 minutes at room temperature. The supernatant was separated with a syringe and added to new Eppendorf tubes. The plant sample was diluted up to 10 mg/mL final concentration for consistent analysis. The final solution was stored for further analysis at 4°C.

#### 2.5.1. Assessment of Polyphenols (TPC, TFC), total antioxidant capacity (DPPH) in in vitro plantlets, ABTS (2,2’-azino-bis (3-ethylbenzothiazoline-6-sulphonic acid) antioxidant assay and Reducing power assay

Folin-Ciocalteu reagent was used to determine TPC activity according to Velioglu’s protocol [23]. In the present experiment, a total of 20 μl sample (10 mg/mL) was loaded into each well of a 96-well plate. Then, 10 times diluted Folin-Ciocalteu (90μL) reagent was poured into the sample wells. After 5 min, the mixture was blended with sodium carbonate (90 μL), resulting in 200 μL. Methanol was the negative control, whereas gallic acid was the positive control. The absorbance was measured at 630 nm after 90 minutes of incubation under a microplate reader. The outcomes were given in milligrams of gallic acid equivalent (GAE) per g. The aluminum trichloride procedure assessed the total flavonoid content. About 20 μL of the sample (10 mg/mL) from each reaction was injected into a well on the microplate. The sample absorbance was determined at 450 nm after 30 minutes using a Biotek microplate reader. The findings were mentioned as milligram quercetin equivalent (QE) per gram.

The sample extract’s potency to detoxify the free radical DPPH was determined as published [24]. The 96-well plate was supplied with different concentrations of the samples; the standard ascorbic acid was used for the positive control group, and absolute methanol for the negative control (10, 5, 2.5, and 1 μL). The wells were then filled with 190 μL, 195 μL, 197.5μL, and 199 μL of DPPH0 solution (4.8 mg/50 mL), respectively, in known methanol concentration. The samples’ final concentrations (1000, 750, 500, and 250 μg/mL) were adjusted. The below-mentioned formula was followed for the determination of free radical scavenging activity.


%scavengingDPPH°freeradical=(1‐AE/AD)×100

Where AE is the solution absorbance when a certain quantity of sample extract is used; AD is the absorbance of DPPH solution without sample extract.

The antioxidant assay, known as the ABTS (2,2’-azino-bis (3-ethylbenzothiazoline-6-sulphonic acid)) radical scavenging assay, was conducted to evaluate the antioxidant activity of *in vitro* plantlet followed by the protocol of [25]. The preparation of the mixture under reaction involved by combining plant extract (1 ml) of each treatment with 1 ml of ABTS+ solution containing K2S2O8 as an oxidizing agent. The absorbance of the reaction mixture was measured at 734 nm to determine the inhibition percentage.

Reducing power assay was conducted following the methodology described by [26]. In this assay, 100 μl of *in vitro* plantlet extract, 1% potassium ferricyanide solution comprising 2.5 mL, was combined with 250 μL of a 0.2 mol/L sodium phosphate buffer solution. This resultant mixture was subjected to incubation at a temperature of 50°C for a duration of 30 minutes. To terminate the reaction, 2.5 mL of 10% trichloroacetic acid was added, followed by centrifugation at 3000 rpm for 10 minutes. Following centrifugation, the supernatant was mixed with 0.5 mL of a 0.1% ferric chloride solution and 2.5 mL of deionized water. Subsequently, the absorbance of this mixture was measured at 700 nm using a spectrophotometer.

#### 2.5.2. Evaluation of enzymatic antioxidants activity

Enzymatic antioxidant defense activities of desired samples were analyzed using the protocol described by [27] with minor alterations. About 2 mL (50 mM) phosphate buffer pH 7.8, 1% Polyvinylpyrrolidone (PVP), and 0.1 mM EDTA solution were used rapidly to produce a homogenate of the mortar-grounded plant material. Further, extracts were centrifuged twice at 12,000 rpm for 15 minutes at 4°C temperature. The activity of Superoxide Dismutase (SOD), Peroxide Dismutase activity (POD), Catalase (CAT), and Ascorbate Peroxidase (APx) activities were determined using a UV–Vis spectrophotometer, according to the Giannopolitis and Ries’ technique [28–30].

### 2.6 Statistical analyses

Statistical analyses for the chosen parameters were performed using SPSS version 16.0. The significance of differences between each parameter was assessed using one-way ANOVA, followed by Duncan’s Multiple Range Test (DMRT) (p ≤ 0.05). Graphs illustrating the various parameters were generated using GraphPad Prism 5.

## 3. Results and discussion

### 3.1. Phytosynthesis and characterization of SeNPs

**Fig 1:**
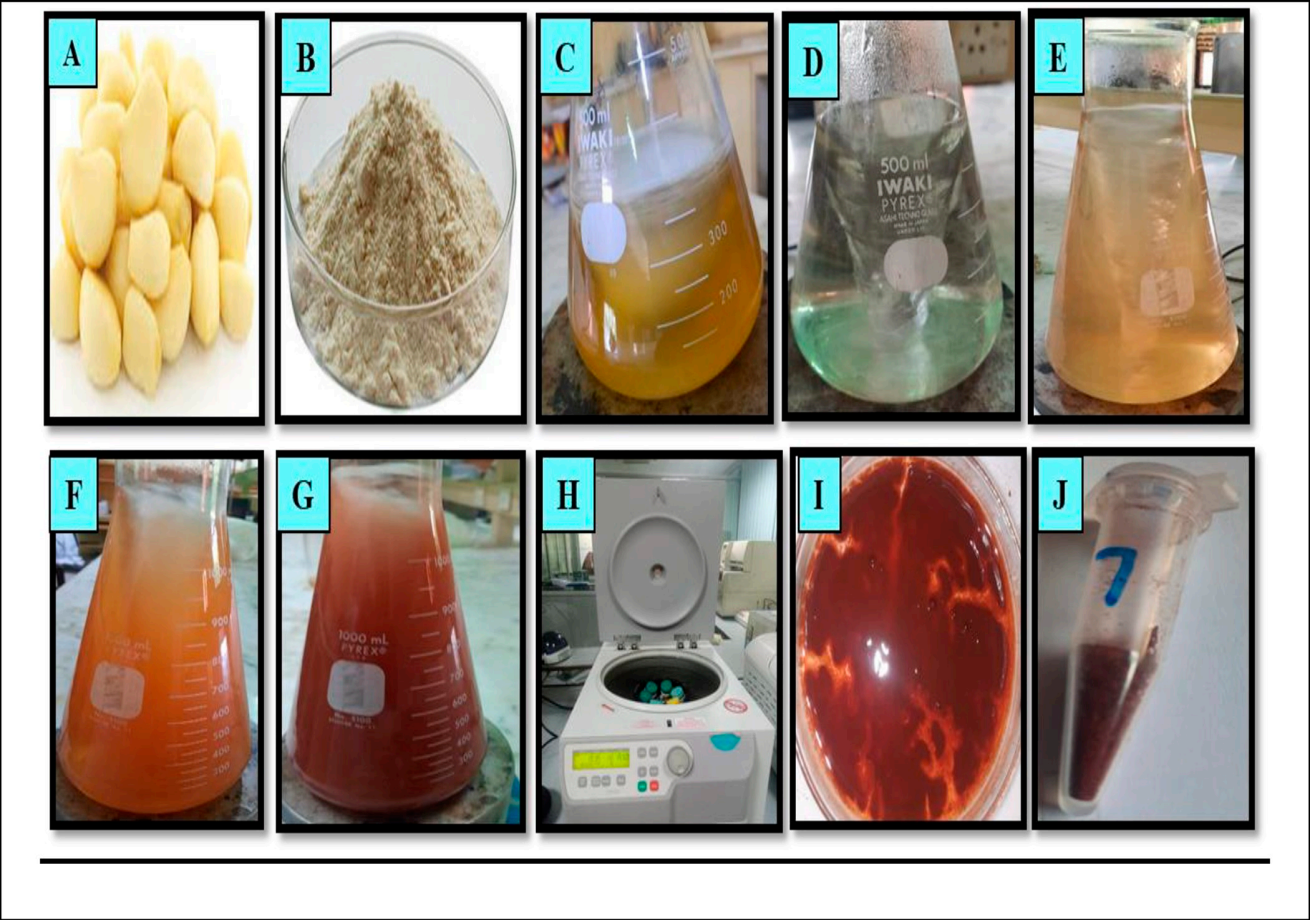
Pictorial Presentation of phytosynthesized selenium nanoparticles using garlic clove extract. Garlic Clove (A), Garlic Powder (B), Garlic extract (C), Sodium Selenite solution (D), the addition of Garlic extract to Sodium Selenite (E), Sequential color changes from light brick (F) to red brick (G), Centrifugation of the mixture solution (H), collected pellet nanoparticles after centrifugation (I), Selenium nanoparticles in dried powder form (J).

Phytosynthesis of nanomaterial is more beneficial than conventional methods, such as chemical or physical methods, due to less toxic, eco-friendly, and cost-effective. In the current research, results confirmed that utilizing garlic clove extract was a suitable reducing, capping, and stabilizing agent for synthesizing SeNPs. The phytosynthesized SeNPs formulation was confirmed through various characterization techniques. The first confirmation of nanoparticle synthesis via visual observation was to change the color to brick red after adding garlic extract (Fig 1).

**Fig 2:**
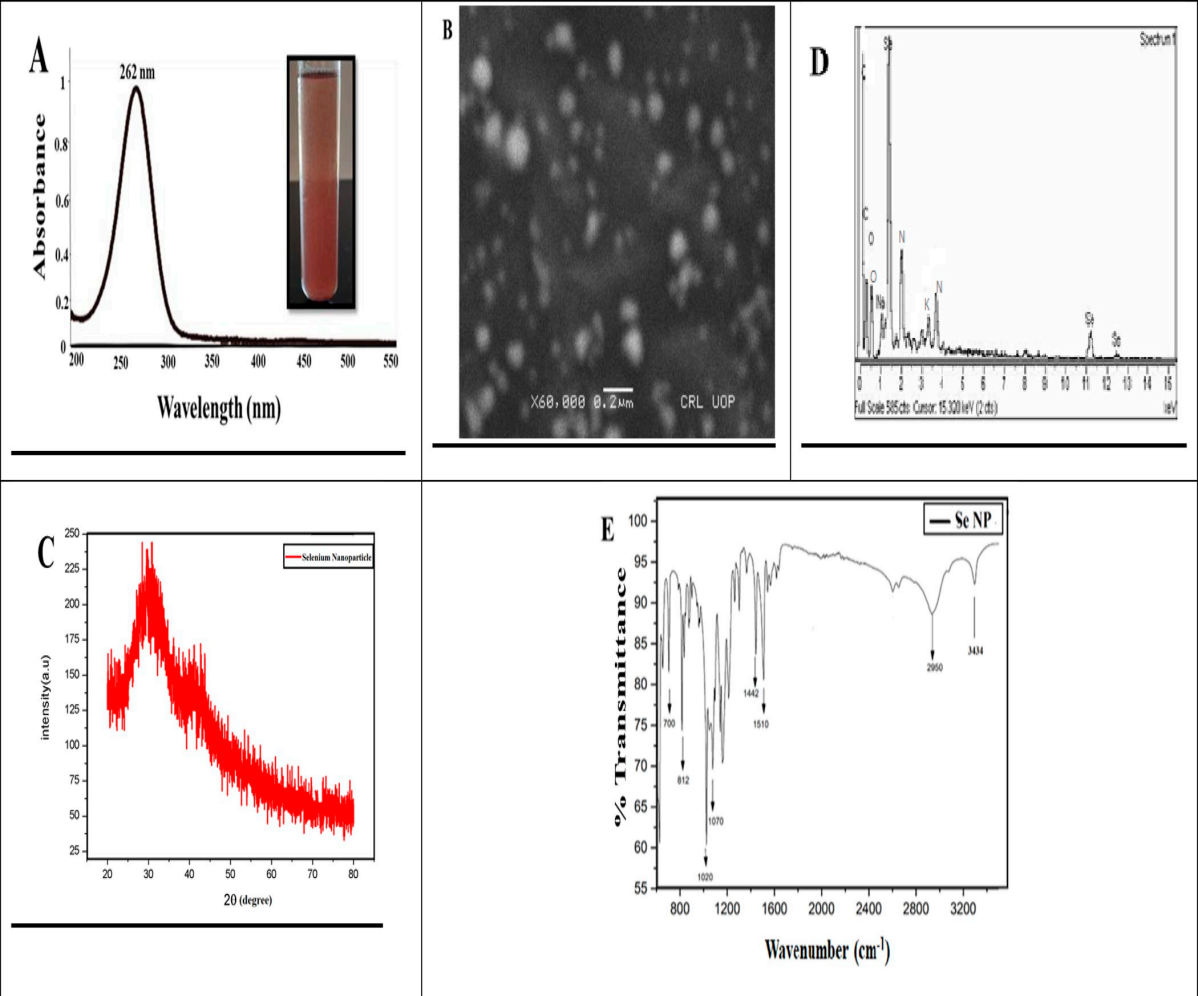
Phytosynthesized selenium nanoparticles using garlic clove extract. Characterization of phytosynthesized Selenium nanoparticles (A to E).

Further, a UV-visible spectrum (200–600 nm) was obtained to confirm the SeNPs synthesis. Characterization peaks of SeNPs were recorded from the 200 nm to 500 nm range. The spectrum showed the peak at 262 nm, demonstrating the features of surface plasma resonance of biosynthesized Selenium NPs Fig 2A.

A similar outcome was also acknowledged by an earlier scientific report in which Garlic clove extract-mediated SeNPs displayed a characterized peak at 260 and 258 nm in the UV visible spectrum [15,31].

To examine the reducing, capping, and stabilizing agent in the SeNPs solution, Fourier Transform Infrared (FT-IR) Spectroscopy was conducted. Results demonstrated that peaks at 3434 cm−1 confirmed the existence of OH and NH groups that are playing an essential role in the NPs synthesis. The peak at 2950 cm−1, 1510 cm−1, and 10220 cm−1 demonstrated the presence of carbon and hydrogen stretching, C = C alkene, and C-O group stretching, which could be the alcohol group certified, and specifies that clove extract makes a bond with sodium Selenite. The absorption peaks 1442 cm−1 and 700 cm−1 2950 cm−1 may be recognized as the existence of aldehyde, carboxylic, oxygen, nitrogen, and amine groups. Our outcomes confirm that, functional groups of garlic cloves extract are significantly involved in the SeNPs formation Fig 2E. In a similar study was also reported by^15^ and showed that, functional groups such as C = C, O-H, N-H, and C = O are responsible for green synthesis. Moreover, a few other reports also resemble our results, concluding that N-H, C-H, and C = O functional groups are involved in forming SeNPs [32]. We may predict that these functional groups play a vital role and might be considered a strong stabilizing and powerful reducing agent during NPs synthesis.

### 3.2. Optimization of Plant growth regulators and Explant types for in vitro Caralluma tuberculata plantlet development

**Fig 3:**
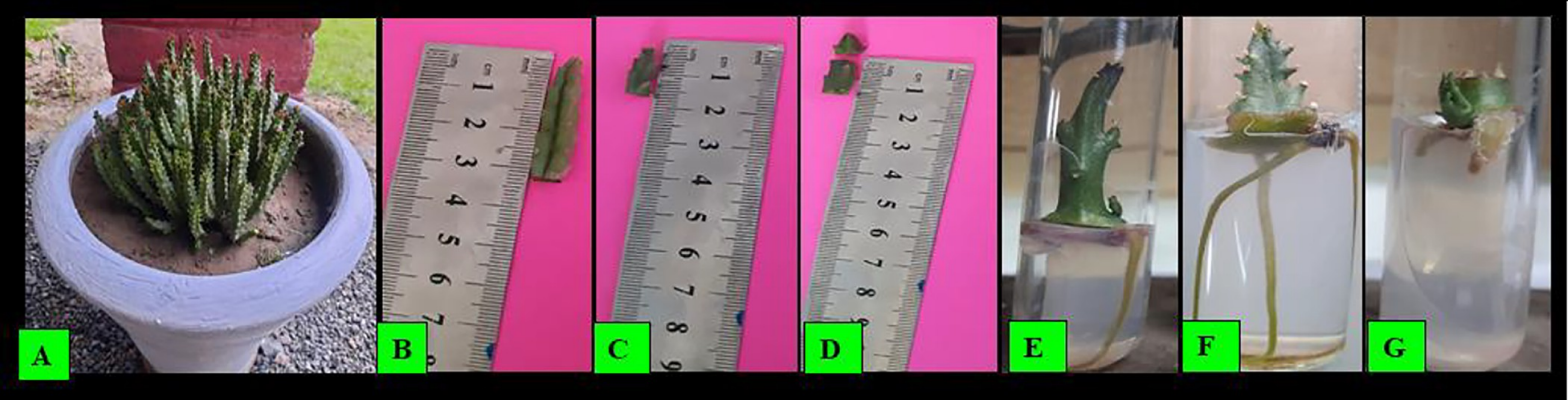
Pictorial presentation of in vitro plantlet induction. (A) *Caralluma tuberculata* grown in pot, (B) Large shoot, (C) Small shoot explant, (D) Cut shoot explant, (E-G) Rooting development in all the applied explants.

In the preliminary investigations, plant growth regulators with various doses and different explant types were employed to induce *C*. *tuberculata* plantlets. When scrutinized the various ranges of auxin (IBA) markedly influenced the rooting induction and the response was noticed in all the applied treatments for *in vitro Caralluma tuberculata* plantlet induction. Using 1 mg/L of IBA promoted early root initiation (7.0±1.7 days), percent response (48.1±4.6%), and average number of roots (3.0±1.0) per plant. However, when the level of IBA was raised from 1 mg/l to 3 mg/l, the negative effect was observed on rooting formation and producing late root initiation (11±2.1), minimum percent response (30±3.6%), and less average number of roots (2.4±0.9) per plant, respectively Table 1 and Fig 3. While explants cultured on MS without PGRs didn’t produce root formation. Auxin is known to be the suitable growth regulator responsible for root induction significantly varies from one plant to the other [33]. According to [34], the influence of auxin on rooting is elicitor at low ranges and inhibitory at higher concentrations. Our findings have similarities with previous reports [35] when they used the same plant growth regulator and observed earlier root initiation at the concentration of 1 mg/l IBA in *Dendrobium* orchid. Similarly [36] also employed auxin to produce *in vitro* rooting in *C*. *tuberculata*.

**Table 1:** Effect of plant growth regulators at various concentrations and different explant on *In vitro* plantlet development.

Treatments	Average No. Days to root initiation of *C*. *tuberculata*			Average (%) response of *C*. *tuberculate*			Average. number of roots/plant of *C*. *tuberculata*		
	Cut shoot	Large shoot	Small shoot	Cut shoot	Large shoot	Small shoot	Cut shoot	Large shoot	Small shoot
MS 0	-	-	-	-	-	-	-	-	-
IBA (0.5 mg/l)	15.2±2.8	14.5±3.1	11.6 ±1.5	28.6 ±3.5	30.4 ±4.5	40.0 ±4.7	2.0 ±0.7	1.6±0.9	2.0 ±1.4
IBA (1 mg/l)	11.0 ±2.6	9.0±2.1	7.0±1.7	37.0±2.8	40.7±3.9	48.1±4.6	2.7±0.6	2.3±0.6	3.0±1.0
IBA (1.5 mg/l)	12.4±1.5	11.8±2.4	10.5±1.5	30.0 ±4.2	35.1±2.8	45.2 ±3.3	2.0 ±1.0	1.8 ±0.7	2.6 ±1.5
IBA (2 mg/l)	14.6 ±1.2	13.3±2.5	10.6±2.0	22.2 ±3.1	18.5 ±2.4	29.6±4.9	1.7±0.6	1.7±0.6	2.3 ±0.6
IBA (2.5 mg/l)	13.4±2.5	15.2±3.4	12.5 ±2.7	28.4 ±2.7	32.0 ±3.8	35.5 ±3.3	1.8 ±0.5	2.0 ±0.9	2.6 ±0.8
IBA (3mg/l)	16.3 ±3.1	14.1±5.1	11.7 ±3.7	24.67 ±3.3	24.67±3.4	30.7±2.6	1.9±0.81	1.7 ±0.7	2.4±0.9

Data were gathered from three replicates and exhibited standard error as determined by Duncan’s Multiple Range Test (DMRT), with a significance level set at p < 0.05

The type of explant has a significant impact on the *in vitro* root induction in *C*. *tuberculata*. In the current study, the comparison of the three explants, namely large shoot, small shoot, and cut shoot, was done for *in vitro* rooting induction. According to our results, the effect of explant types on root initiation, percent response, and number of roots in *C*. *tuberculata* showed highly significant differences in the culture media. Small shoot explants performed better and responded well than large shoot and cut shoot explants for root induction Table 1 and Fig 3.

Our results compare with the number of reporters [19,37] where they reported that the source of explants, explants type, and age are essential factors in determining *in vitro* responses. This observation indicates that there could be variability in the levels of endogenous hormones or their responsiveness across organs. Furthermore, previous research [38–40] has documented the impact of explant type on achieving favorable outcomes in tissue culture for various crops.

### 3.3. Selenium nanoparticles mediated micropropagation and growth parameters of Caralluma tuberculata

**Fig 4:**
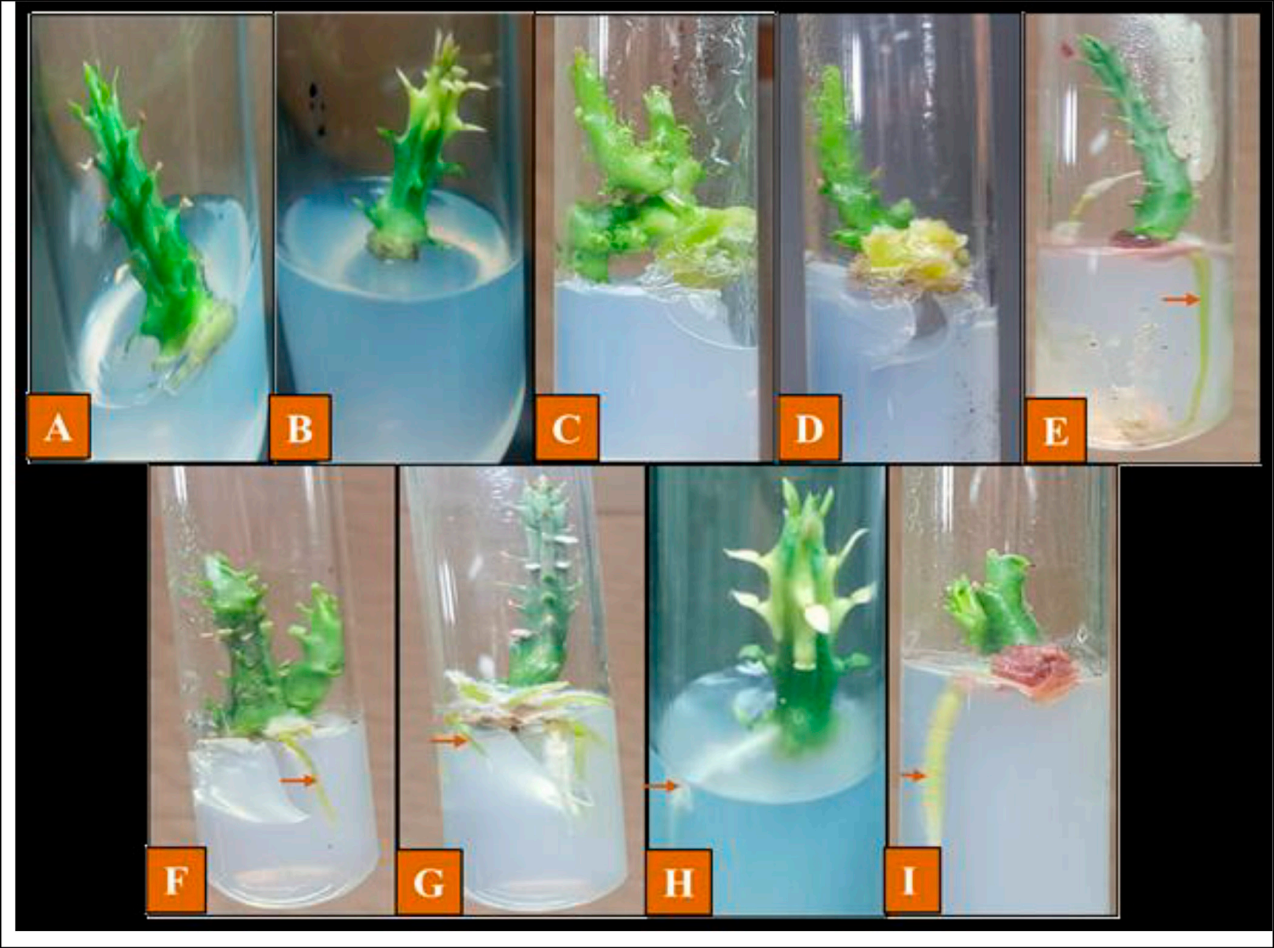
Effect of various concentrations of selenium nanoparticles either alone or in combination with PGRs on *in vitro* plantlet development. (A) MS media along with 50 ug/L SeNPs, (B) MS media along with 100 ug/L SeNPs, (C) MS media along with 200 ug/L SeNPs, (D) MS media along with 400 ug/L SeNPs, (E) MS media along with 50 ug/L SeNPs + IBA 1mg/L, (F) MS media along with 100 ug/L SeNPs + IBA 1mg/L, (G) MS media along with 200 ug/L SeNPs + IBA 1mg/L, (H) MS media along with 400 ug/L SeNPs+ IBA 1mg/L.

To investigate the impact of biogenic selenium nanoparticles on *in vitro* plantlet development, it was demonstrated that the effect of SeNPs was dose-dependent. The cultures obtained from MS media augmented with 100 μg/L and PGRs (1 mg/L IBA) produced the earliest root initiation (4.6±0.24 days), highest root frequency (68.21±5.12), number of roots (6.3±1.81), maximum fresh weight (710±6.01 mg) and dry weight (549±6.77 mg) respectively (Table 2 and Fig 4).

**Table 2:** Effect of phyto-mediated Selenium nanoparticles on *in vitro* rooting development in *Caralluma tuberculata*.

Treatments	Days to root initiation	Frequency of root induction	Max number of root induction	FW (Full Plant) mg	DW (Full Plant) mg
**SeNPs (50 μg/l)**	-	-	-	653.21±7.31	488.04±4.09
**SeNPs (100 μg/l)**	-	-	-	577.45±6.05	439.87±3.51
**SeNPs (200 μg/l)**	-	-	-	695.65±8.26	539.24±5.62
**SeNPs (400 μg/l)**	-	-	-	605.91±5.83	447.02±3.48
**IBA(1mg/l) (Control)**	8.3±1.20	44.35±3.2	2.8±1.23	541.13±4.21	403.24±6.13
**SeNPs (50μg/l)+ IBA (1mg/l)**	7.5±1.56	53.26±4.45	2.4±1.01	630.72±6.24	490.44±4.01
**SeNPs (100 μg/l)+ IBA (1mg/l)**	4.6±0.98	68.21±5.12	6.3±1.8	710.18±6.01	549.89±6.77
**SeNPs (200 μg/l)+ IBA (1mg/l)**	8.9±1.27	51.33±3.37	1.6±1.3	620.11±4.71	463.16±4.53
**SeNPs (400 μg/l)+ IBA (1mg/l)**	11.3±2.13	43.14±3.26	1.2±0.9	442.52±4.23	298.88±3.43

Data were gathered from three replicates and exhibited standard error as determined by Duncan’s Multiple Range Test (DMRT), with a significance level set at p < 0.05.

Similar outcomes were reported by [41] where SeNPs boosted tobacco root development at the optimal range. It was justified that lower concentrations of SeNPs elicit the induction of roots, cause less phytotoxicity, and have a tremendous effect on the morphological and microstructure profiling of the root [42]. Consistent with our outcomes, the application of SeNPs stimulated root development in *Nicotiana tabacum* by prompting auxin fabrications [43]. In a study conducted by Darwesh and colleagues in 2023, an investigation was undertaken to assess the impact of silver, chitosan, and selenium nanoparticles on the *in vitro* growth of plantlets from three distinct olive cultivars: Koroneiki, Picual, and Manzanillo. The introduction of nanoparticles into the growth medium yielded noteworthy effects on the proliferation and growth rate of *in vitro* olive plants [44]. Therefore, we may suggest that phyto-mediated SeNPs elicited the rooting development of *C*. *tuberculata* by interfering with endogenous auxin pathways. The synthesis of growth regulators like auxin is also accelerated by the optimal range of Se, which affects root architecture and improves root development [45].

According to [46], the mechanistic approach of selenium to root development in plants is based on stimulating the auxin biosynthesis pathway. This might be because Se escalated the gene expression involved in manufacturing auxin. In another report, they justified the essential role of Se that expresses the nutrient transporter genes such as SULT1, SULT2, and SULT3 significantly and provide sufficient nutrients for plant growth and development. Augmentation of Se NPs at an optimal level can be beneficial to increase the capacity of nutrient uptake by explant and accelerate the rooting growth and development.

The supplementation of selenium nanoparticles at higher levels (400 ug/L), along with plant growth regulators (PGRs), in the culture media resulted in delayed root initiation (11.3±0.13), decreased root frequency (43.14±3.26), inhibited rooting development (1.2±0.9), and reduced fresh weight (442.52± mg) and dry weight (298.88±2.43 mg) Table 2 and Fig 4.

These adverse effects are likely caused by the toxicity induced by higher concentrations of nanoparticles, which have been found to negatively impact the growth of plant cultures in previous studies [47]. Our survey on *Caralluma tuberculata* plantlets also demonstrated similar findings, indicating that high concentrations of selenium nanoparticles adversely affected their *in vitro* growth. This might be attributed to the inhibition of plant hormone fabrications and reduced action of ROS scavenging enzymes [48]. Additionally, excessive absorption of selenium by the explants may disrupt biochemical processes, leading to the production of harmful substances like superoxide and ethylene. These substances can stimulate the chlorophyllase enzyme, which can mutilate the chloroplast membrane and ultimately hinder the growth of plant cultures [47,49]. The injurious properties of higher doses of NPs on plant cultures have been observed and reported in several studies [50,51]. Furthermore, numerous scientific reports [18,47,52,53] have highlighted the diverse impact of various nanoparticles on plants, which can either enhance or reduce plant growth and productivity. Zaka and colleagues [54] conducted a comparative analysis involving silver nanoparticles (AgNPs), gold nanoparticles (AuNPs), and copper nanoparticles (CuNPs) in the context of *Eruca sativa* Mill. They found that the utilization of AgNPs resulted in enhanced shoot and root growth. In another investigation involving Musa spp., it was verified that the incorporation of AgNPs into the growth medium led to an increase in the number of roots, their length, root growth and fresh weight to dry weight ratio [55]. It is worth noting that the MS media containing nanoparticles but without PGRs did not induce root formation Table 2.

### 3.4. Physiological profiling of selenium nanoparticles mediated micro propagated Caralluma tuberculata plants

**Fig 5:**
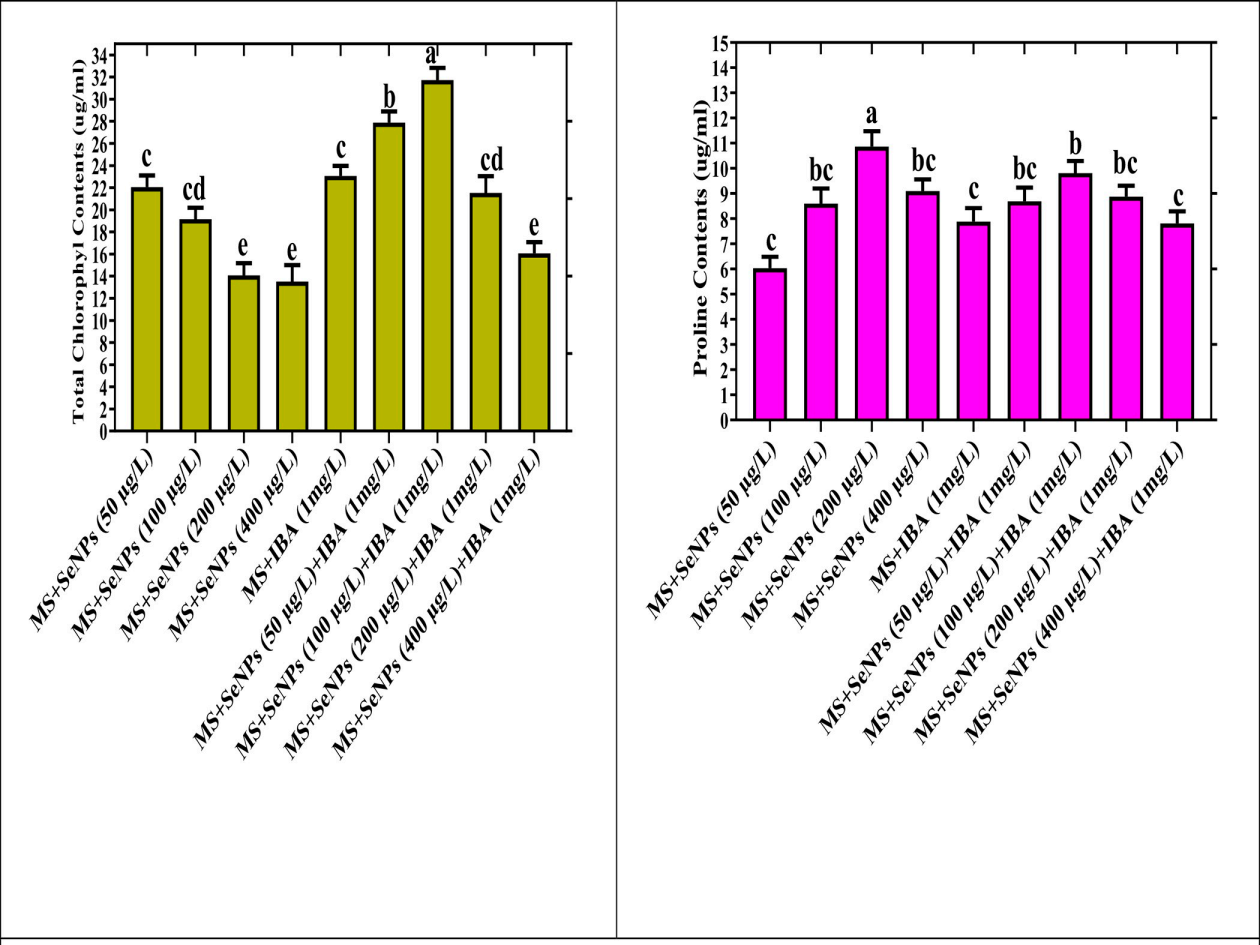
An assessment was undertaken to examine the biochemical profiling in response to various doses of SeNPs treatments of *Caralluma tuberculata*. The dataset presents the mean values obtained from triplicate experiments, along with the standard error denoted by ±, with a statistical significance threshold set at p < 0.05. The parameters investigated encompass the Total Chlorophyll Contents and the Total Proline Contents.

When plant cultures are grown in a stressful environment, a surplus of oxidative species is generated, negatively affecting the photosynthetic process. Fascinatingly, the synthesis of chlorophyll and other photosynthetic pigments can be accelerated at optimal doses of Se, which might be less toxic to destroy chloroplasts [56]. In our study, SeNPs (50 μg/L) along with IBA (1 mg/L) had a promotive effect on total chlorophyll content (32.66±3.46 μg/ml) in the culture at a feasible level in the *Caralluma tuberculata in vitro* cultures Fig 5.

In contrast, the use of a high concentration of selenium nanoparticles (SeNPs) at 400 μg/L had a clear negative impact on the quality of the plantlets. It resulted in a significant reduction in the total chlorophyll content (14.28±2.35 μg/ml) Fig 5.

Previous studies have suggested that selenium plays a role in regulating the photosynthesis antenna complex by restructuring it to enhance energy absorption and protect plants from oxidative stress [57,58]. At lower concentrations, selenium application has been found to increase chlorophyll synthesis and stimulate the photosynthetic machinery acknowledged by [57,59]. The incorporation of nanoparticles (NPs) into culture media has been observed to enhance photosynthetic activities by influencing critical physiological processes [26]. A study by Parida and Das [60] reported that the application of NPs to cultures resulted in an improved chlorophyll ratio, indicating heightened activity of PS-I and PS-II, potentially facilitating regeneration. Additionally, there is evidence suggesting that both iron nanoparticles (FeNPs) and zinc oxide nanoparticles (ZnONPs) have been shown to upregulate the gene expression of enzymes crucial for photosynthesis, thereby contributing to its enhancement [61]. Similarly, ZnONPs have been found to promote plant growth by influencing the electron transfer chain, increasing enzymatic antioxidants, reducing ion leakage, and enhancing the Hill reaction [62].

During an unfavorable environment, an osmoprotectant called proline protects the plant from stress by scavenging the ROS species and creating favorable conditions for plant growth. Our study observed an increase in the total proline content in all the treatment groups. The highest concentration of SeNPs (200 μg/L) alone resulted in the maximum proline content (10.5±2.22 μg/ml) compared to other treatments. While applying the SeNPs along with PGRs gradually declined, the proline contents in the cultures. Consistent with our results, a previous study has been reported to enhance proline contents in the *Oryza sativa* and *Arabidopsis thaliana* due to exposure to AgNPs and copper oxide NPs [63]. The stress caused by Se NPs led to an increased accumulation of proline, as observed in the present study, which could aim to protect the cultures from severe stress conditions.

### 3.5. Non-enzymatic and enzymatic antioxidant profiling of selenium nanoparticles mediated micro propagated Caralluma tuberculata plants

Several previous studies have explored the secondary metabolites and antioxidant potential of *Caralluma* species [4,64,65]. However, there is a need for an optimized method to achieve the feasible production of polyphenols with potent antioxidant properties in *Caralluma tuberculata*. To address this, we subjected *C*. *tuberculata* explants to stress conditions by incubating them in the presence of selenium nanoparticles (SeNPs) alone or combination with PGRs across various concentrations in MS media. We measured the total phenolic content (TPC), total flavonoid content (TFC), and DPPH free radical scavenging activity in the established *in vitro* plantlets under SeNPs treatments.

**Fig 6:**
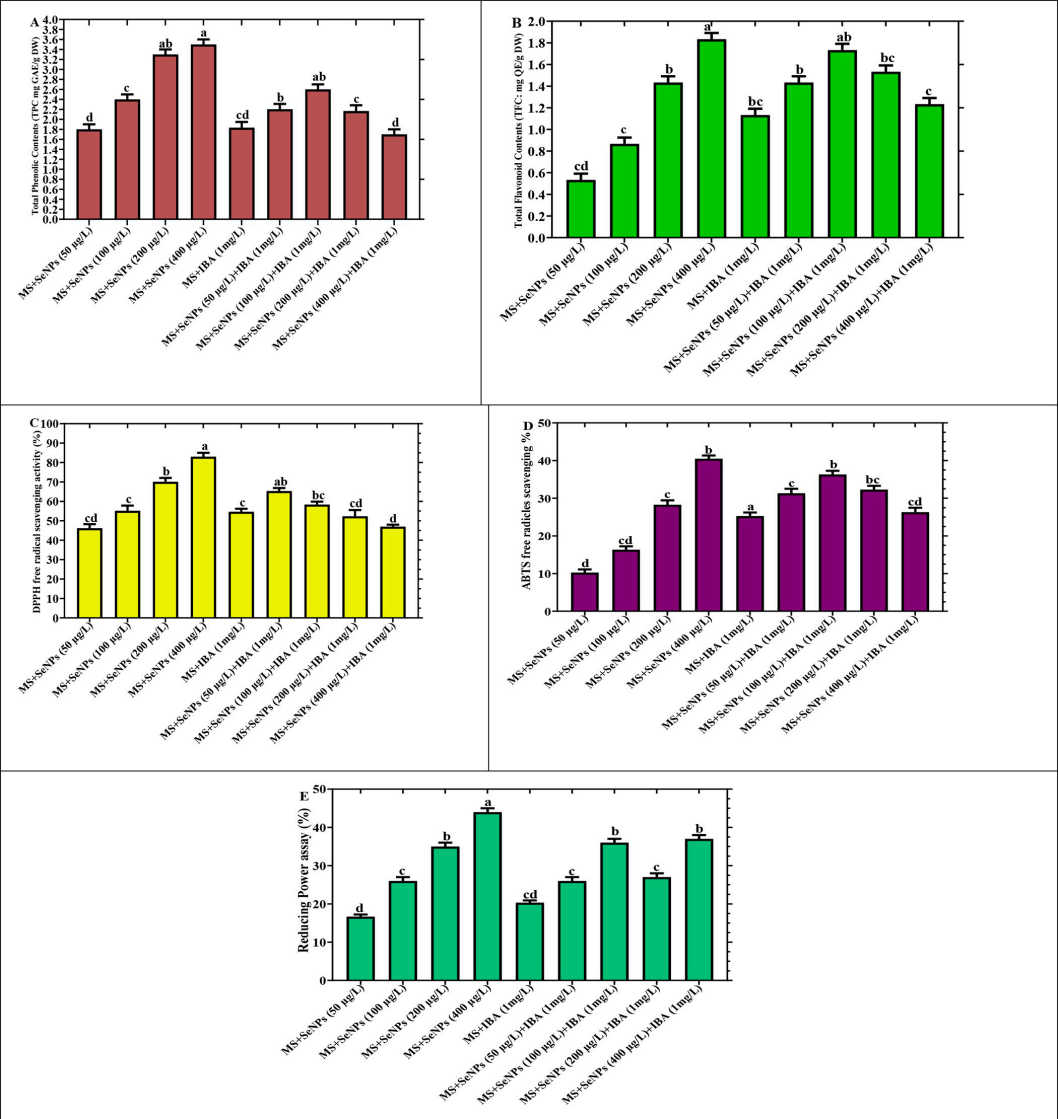
The levels of non-enzymatic antioxidant elements in *Caralluma tuberculata* were evaluated in response to various concentrations of selenium nanoparticles (SeNPs) treatments. The data presented are the average values of triplicate measurements, with standard errors indicated, at a significance level of p < 0.05. (A) Total phenolic content (TPC), (B) Total flavonoid content (TFC), (C) free radical scavenging activity (DPPH), (D) ABTS (2,2’-azino-bis (3-ethylbenzothiazoline-6-sulphonic acid) antioxidant assay, (E) Reducing power assay.

Our results showed that when SeNPs were tested alone, they enhanced the production of bioactive metabolites at higher levels. The maximum accumulation of TPC (3.0 mg GAE/g), TFC (1.8 mg QAE/g), and DPPH free radical activity (82%) was observed in the culture established with 400 μg/L SeNPs in the MS media Fig 6.

However, when the plant samples were exposed to the same level of SeNPs (400 μg/L) in combination with PGRs, no substantial fabrications in these compounds were detected. In comparison, plants grown in a stress-free environment (control) accumulated relatively lower levels of TPC, TFC, and DPPH free radical scavenging activity (1.6 mg GAE/g, 0.4 mg QAE/g, 54%) Fig 6A–6C.

Similar increases in polyphenol content have been reported in plant cultures treated with other nanoparticles [18,66,67]. However, the mechanistic effects of nanomaterials on the metabolomics profile of *in vitro* plants have not been well studied or understood.

One possible explanation is that nanoparticles can induce oxidative stress in plants, prompting them to respond by modifying their biochemical and antioxidant processes. In this stressful environment, the equilibrium between the generation of oxidative spp and their amputation by scavengers is disrupted, resulting in significant damage to carbohydrates, lipids, proteins nucleic acids, and eventually, this damage can cause plant cell death. Furthermore, it has been suggested that reactive oxygen species (ROS) can harm chlorophyll synthesis, leading to cellular damage. This damage, in turn, can trigger secondary metabolism as a response to cope with the injury [68]. Several studies have demonstrated that various nanoparticles (Cerium, gold, silver, zinc, Titanium, etc.,) can stimulate the generation of ROS, which subsequently promotes bioactive compounds to counteract the harmful impacts on plantlet improvement [4,18,66,67,69]. The positive impact of nanoparticles on enhancing the production of bioactive compounds in plants has been substantiated by various studies. For instance, *Artemisia anuua* was validated by [70], *Hypericum perforatum* by [71], and *Glycyrrhiza glabra* by [72]. In addition, silver and gold nanoparticles have served as elicitors in the cultivation of *Prunella vulgaris* [73], Stevia glycosides [74], *Cucumis anguria* [75]. Recent scientific findings have revealed significant alterations in the metabolite profile of *Arabidopsis thaliana* when exposed to silver nanoparticles [76].

Current findings indicate that when *C*. *tuberculata* was exposed to Selenium nanoparticles (SeNPs), there was an improvement in the fabrication of secondary compounds and an increase in antioxidant activity.

The study underscores the remarkable radical scavenging capabilities of *Caralluma tuberculata* plantlets cultivated *in vitro*, highlighting their effectiveness in addressing ABTS+ oxidation. The ABTS+ assessment serves as a measure of antioxidants’ ability to counteract the oxidative agents produced by ABTS+. Within the scope of this research, it was observed that the percentage of scavenging efficacy reached its peak at 40.22% when plant cultures were exclusively exposed to SeNPs at a concentration of 400 μg/L alone. In contrast, when the same SeNPs level (400 μg/L) was combined with plant growth regulators (PGRs), the scavenging activity dropped to 24.23%. Additionally, plantlets grown in an unperturbed environment (control group) displayed relatively lower levels of free radical scavenging activity Fig 6D. These findings are consistent with previous research [26], which demonstrated that *Caralluma tuberculata* shoot extracts exhibited the highest reduction in ABTS+ radical species. This suggests that these extracts selectively combat reactive oxygen species (ROS) within normal cells, shielding them from oxidative stress [60]. This investigation’s results reveal the significant medicinal value of *Caralluma tuberculata* plantlets and their rich phytochemical composition, characterized by potent antioxidant properties. Their ability to intercept radical chain reactions is particularly noteworthy. Similarly, the extract from *C*. *flava* demonstrated notable radical scavenging attributes in ABTS assays, positioning *C*. *flava* as a promising source of both enzymatic and non-enzymatic antioxidants [61].

The presence of antioxidants is closely associated with their reducing properties, involving the donation of hydrogen atoms, interruption of free radical chains, and exertion of antioxidative effects [62]. Consequently, it is reasonable to infer that *C*. *tuberculata* possesses a high antioxidant content, which interferes with radical chain reactions by engaging free radicals to establish stability. *In vitro* Plantlets treated with SeNPs at a concentration of 400 μg/L exhibited a reducing power activity of 43.21%. In comparison, the reducing power assay yielded values of 37.23% for SeNPs (400 μg/L) combined with IBA (1mg/L). Conversely, plantlets treated only with IBA (1mg/L) displayed a scavenging potential of 20.56% Fig 6E. The robust antioxidant profile of *C*. *tuberculata* is further elucidated by its performance in the reducing power assay, which demonstrated a concentration-dependent effect. Moreover, the study delved into the reducing power of *in vitro* culture extracts from five distinct Ocimum species (*Ocimum sanctum*, *Ocimum kilimandscharicum*, *Ocimum gratissimum*, *Ocimum basilicum*, and *Ocimum americanum*) notable activity was observed, primarily attributed to the enhanced accumulation of polyphenols within the extracts [70].

**Fig 7:**
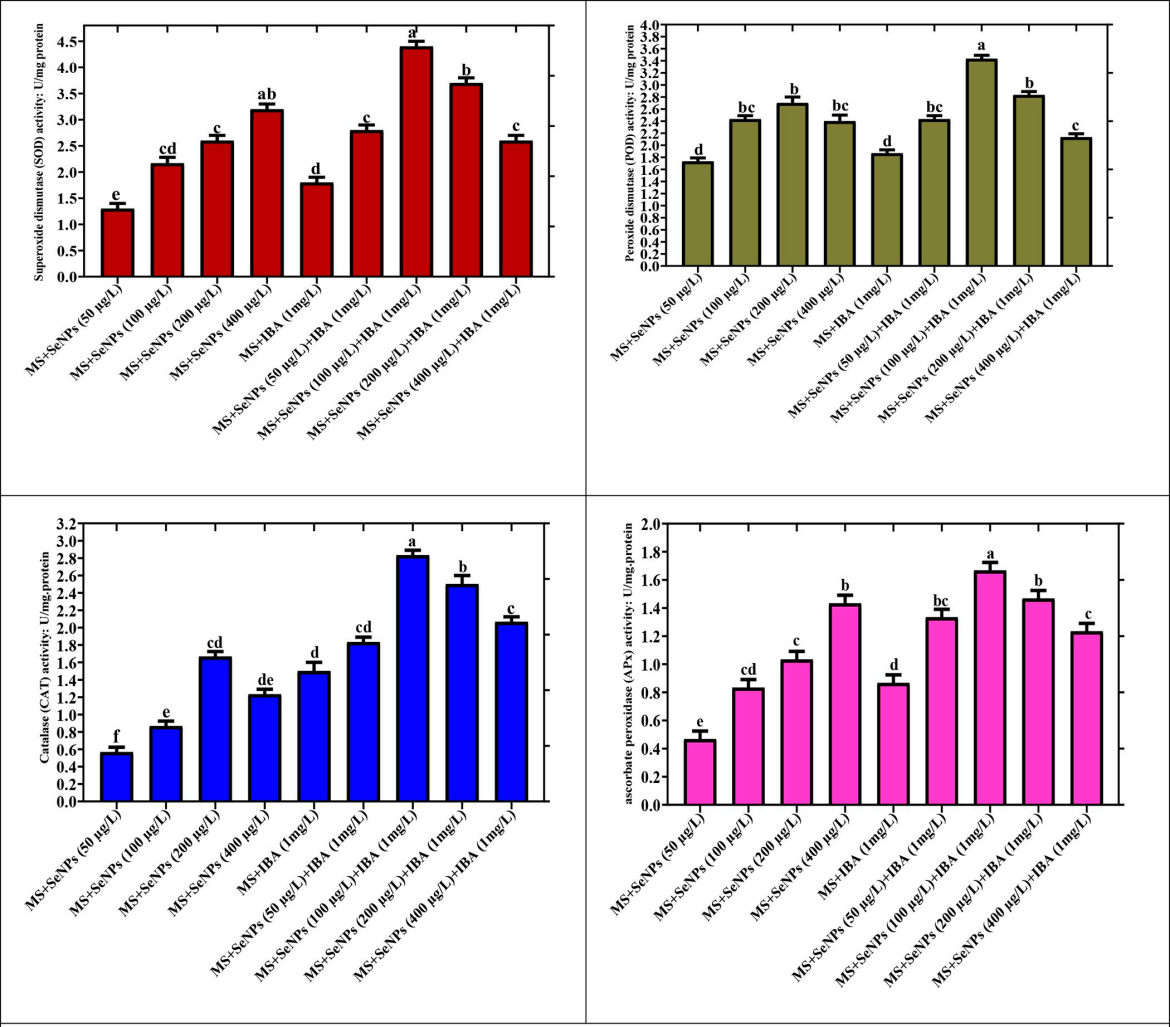
The enzymatic antioxidant elements in *Caralluma tuberculata* were assessed in relation to the effects of different concentrations of selenium nanoparticles (SeNPs) treatments. The data provided represents the average values of triplicate measurements, with standard errors indicated, at a significance level of p < 0.05. (A) superoxide dismutase (SOD) activity, (B) peroxidase (POD) activity, (C) catalase (CAT) activity, and (D) ascorbate peroxidase (APx) activity.

Plants employ robust enzymatic machinery to combat reactive oxygen species (ROS). In this investigation, the actions of crucial antioxidant enzymes, (SOD, POD, CAT, and APx) were assessed in *in vitro* plantlets treated with various doses of SeNPs, either solitary or in amalgamation with 1 mg/l IBA. Among all the treatments, the combination of SeNPs (100 mg/L) with PGRs boosted SOD, POD, CAT, and APx (4.4, 3.3, 2.8, and 1.6 U/mg protein, respectively) potential in the *in vitro* plantlets at considerable level Fig 7.

On the other hand, there was no significant improvement in antioxidant enzymes observed in cultures treated solely with SeNPs at 50 and 100 mg/L concentrations, showing lower values for SOD: POD: CAT and APx (1.2 U/mg, 1.7 U/mg, 0.5 U/mg, and 0.43 U/mg, respectively). Compared to the control treatment, moderate levels of enzyme activities were detected at higher amounts of SeNPs (200 and 400 μg/L) when used alone in the plantlet. These findings suggest that combining SeNPs at optimal concentrations with PGRs proved more effective in promoting the manufacturing of antioxidant enzymes.

Similar to other investigations conducted by [77], the impact of AgNPs at various ranges on the enzymatic antioxidant profiling of *Brassica juncea in vitro* plants was evaluated. Their findings indicated that enhancing the supplementation of AgNPs in the growth media elicited enzymatic antioxidant potential. Consistent with these results, our study validated that a higher dose of SeNPs also stimulated SPD activity in the plantlet relative to a lower dose. Additionally, [78] stated that exposure of tomato plants to elevated doses of NPs boosted SOD activity.

Applying an optimal dose of SeNPs in the culture media enriched the activity of antioxidant enzymes in the *in vitro* plants. When plants are exposed to nanoparticles, a sequence of occurrences cascades within the plant cells. This leads to an oxidative burst and oxidative species (ROS) produced in the plant cells’ nearby environment. The secondary metabolites act as scavengers for the oxidative species, and as a consequence, cell division and cellular responses are modulated to accomplish optimal plantlet growth. Amidst the free radical scavenging enzymes, CAT and APX play a crucial role in scavenging ROS and mitigating oxidative stress [79].

In recent years, selenium (Se) has emerged as a promising candidate for prompting the antioxidant profile of plants, as recognized by [80]. While Se is commonly used in agriculture, its potential benefits in micropropagation have not yet been fully explored [81]. Additionally, the application of Se for the development of *in vitro Caralluma tuberculata* plantlets has not yet been acknowledged. However, recently, [82] investigated the impact of photosynthesized selenium nanoparticles and light regimes on *in vitro Caralluma tuberculata* callus cultures. Results showed that, cultures grown on Murashige and Skoog (MS) media containing SeNPs (100 μg/L), in a dark environment for two weeks, and then transferred into normal light, accumulated maximum polyphenols and enzymatic antioxidant activities. In another scientific investigation carried out by [25], and conducted an experiment involving the application of SiO2NPs on in vitro cultures of Tagetes erecta L. The results indicated a notable increase in the levels of polyphenolic compounds at higher nanoparticle concentrations, in contrast to lower nanoparticle concentrations. In the current project, we observed an affirmative consequence of Se nanoparticles (SeNPs) on the activity of necessary antioxidant enzymes, including SOD, POD, CAT, and APX. Notably, the increase in enzymatic activities was strongly associated with the growth of *in vitro C*. *tuberculata* plantlets. These findings align with previous research suggesting that SeNPs can promote plant growth by interacting with the antioxidant system.

**Fig 8:**
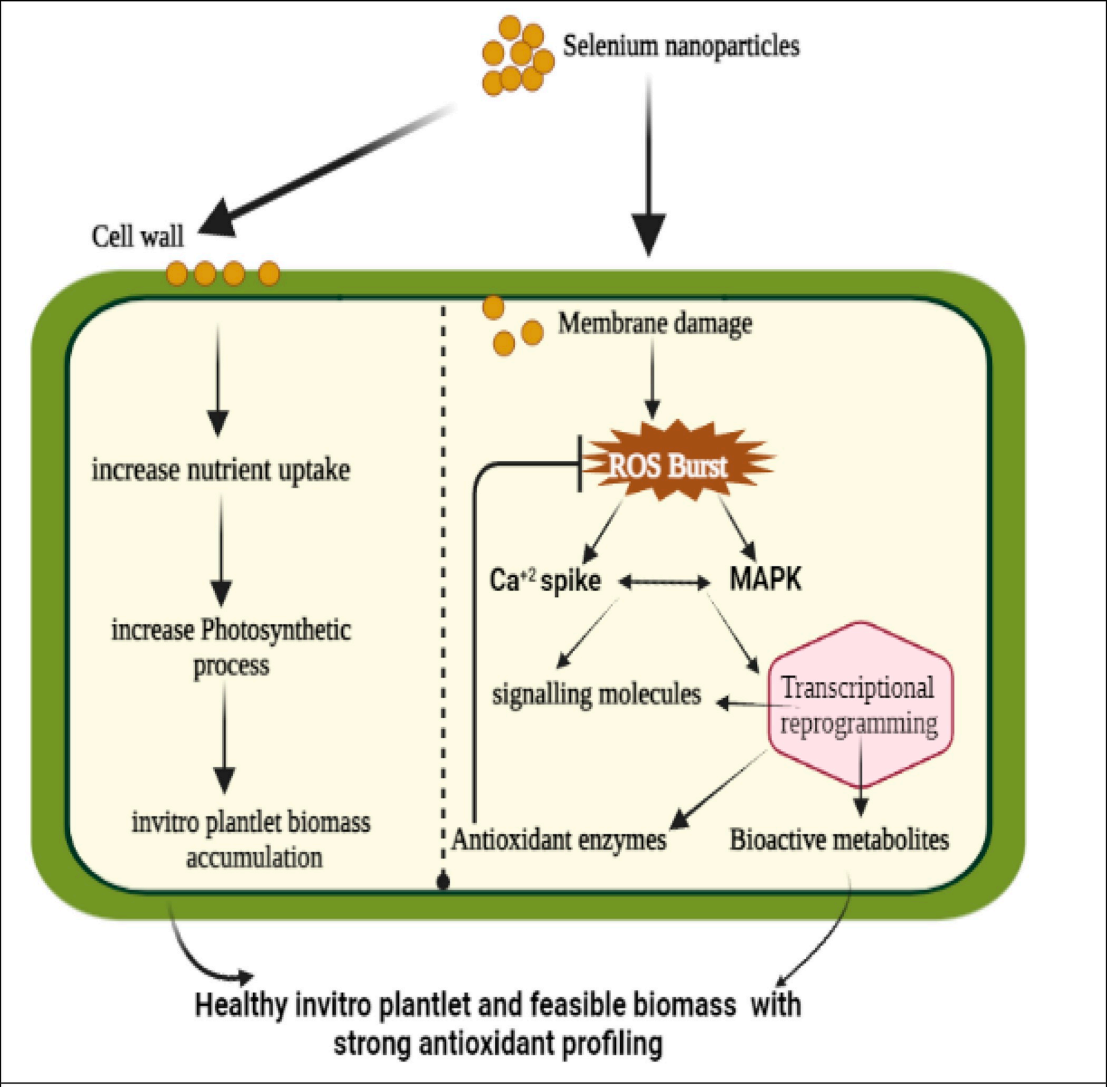
Possible mechanism of phyto mediated nanoparticles (SeNPs) effect on *in vitro* plant cell.

Literature explores the potential mechanisms behind enhancing growth and biomass accumulation observed with the administration of Selenium Nanoparticles (SeNPs). The introduction of SNPs has been found to significantly increase nutrient uptake, promoting the growth of *in vitro* plantlet development. This augmentation in growth can be attributed to the stimulation of photosynthetic activity, leading to biomass formation. Additionally, SeNPs have been observed to initiate a cascade of ROS-mediated MAPK signaling and a surge in calcium levels. These effects are believed to enhance the production of secondary metabolites, serving as a defense mechanism against oxidative stress Fig 8.

The application of SeNPs results in an improved rooting development and fabrications of secondary metabolites in *Caralluma tuberculata in vitro*.

## 4. Conclusions

In this paper, we scrutinized the impact of phytosynthesized selenium nanoparticles (SeNPs) on the *in vitro* rooting development, physiological and antioxidant profile of *C*. *tuberculata*. Various characterizations data have confirmed that garlic clove extract is a suitable agent for reducing, capping, and stabilizing the synthesis of SeNPs. Additionally, the use of SeNPs as nanoelicitors has shown significant benefits in promoting root growth and the production of pharmacologically active metabolites in *C*. *tuberculata*. When SeNPs were added to MS media at a concentration of 100 μg/L along with IBA (1 mg/L), it notably stimulated root development and other physical attributes. However, when higher levels of selenium nanoparticles (400 ug/L) were combined with IBA (1 mg/L), root growth was delayed. For the efficient production of total chlorophyll content and total proline content, cultures treated with SeNPs at concentrations of 50 μg/L and 200 μg/L, in conjunction with IBA (1 mg/L), proved to be suitable. Moreover, the application of SeNPs at a concentration of 200 μg/L alone was found to induce the biosynthesis of polyphenols (TPC, TFC), which are closely associated with the essential total antioxidant potential (DPPH, reducing power assay, ABTs assay) in the cultured samples compared to the control group. Furthermore, higher quantities of antioxidant enzymes (SOD, POD, CAT, APx) were observed in the cultures treated with SeNPs at 100 μg/L and IBA at 1 mg/L. These results suggest that the utilization of SeNPs holds great promise as an effective approach for the large-scale commercial production of *in vitro C*. *tuberculata*, ensuring a sufficient supply of secondary metabolites.

## Abbreviations

SeNPs: Selenium Nanoparticles
NPs: Nanoparticle
TPC: Total phenolic contents
TFC: Total flavonoid contents
SOD: Superoxide Dismutase
POD: Peroxide Dismutase
APx: Ascorbate Peroxidase
CAT: Catalase
DM: Diabetes Mellitus
SEM: Scanning Electron Microscopy
EDX: Energy Dispersive X-ray Spectroscopy
FTIR: Fourier Transform Infrared Spectroscopy
XRD: X-ray Diffraction
FW: Fresh Weight
MS: Murashige and Skoog
ROS: Reactive Oxygen Species
dH2O: Distilled water
PGRs: Plant Growth Regulators
IBA: Indole-3-butyric acid
TCC: Total Chlorophyll Content
TPC: Total Proline Content
